# Innate Pulmonary Phagocytes and Their Interactions with Pathogenic *Cryptococcus* Species

**DOI:** 10.3390/jof9060617

**Published:** 2023-05-27

**Authors:** Brittney N. Conn, Karen L. Wozniak

**Affiliations:** Department of Microbiology and Molecular Genetics, Oklahoma State University, 307 Life Science East, Stillwater, OK 74078, USA; brittney.jackson@okstate.edu

**Keywords:** *Cryptococcus neoformans*, innate immune response, macrophages, dendritic cells, neutrophils

## Abstract

*Cryptococcus neoformans* is an opportunistic fungal pathogen that causes over 180,000 annual deaths in HIV/AIDS patients. Innate phagocytes in the lungs, such as dendritic cells (DCs) and macrophages, are the first cells to interact with the pathogen. Neutrophils, another innate phagocyte, are recruited to the lungs during cryptococcal infection. These innate cells are involved in early detection of *C. neoformans*, as well as the removal and clearance of cryptococcal infections. However, *C. neoformans* has developed ways to interfere with these processes, allowing for the evasion of the host’s innate immune system. Additionally, the innate immune cells have the ability to aid in cryptococcal pathogenesis. This review discusses recent literature on the interactions of innate pulmonary phagocytes with *C. neoformans*.

## 1. Introduction

*Cryptococcus neoformans* is an encapsulated fungal pathogen that primarily causes infections in immunocompromised individuals. It is found throughout the environment in soil, decaying wood, and bird excrement [1,2]. Infection by the pathogen occurs via the inhalation of the basidospores or yeast from environmental reservoirs into the pulmonary alveoli, where they initially interact with the resident host innate immune cells [2,3,4]. These cells include subsets of macrophages and dendritic cells (DCs), which are involved in the early recognition as well as removal and clearance of the cryptococcal infection [5,6,7,8,9,10,11,12,13,14]. Following initial infection, neutrophils are recruited to the lung [15,16,17]. Recent studies have found both beneficial and damaging abilities of these innate immune cells during cryptococcal pathogenesis. They have examined the potential of intracellular growth of *C. neoformans* in macrophages and neutrophils, as well as the use of anti-cryptococcal abilities of DCs in host defense [12,17,18,19,20,21,22,23,24].

In this review, recent literature on the cryptococcal interactions between DCs, macrophages, and neutrophils during infection will be discussed, including a closer look at the potential damaging ability of these cells to the host during cryptococcal pathogenesis.

## 2. Dendritic Cells

Dendritic cells (DCs) function as one of the first types of immune cells to encounter airway pathogens. DCs are resident cells of the lungs and serve as sentinels of the immune system, recognizing antigens as they are inhaled into the lungs (reviewed in [25]). As phagocytes, DCs have the ability to recognize, engulf, and destroy these pathogens. Additionally, they can circulate to regional lymph nodes for antigen presentation to naïve T cells, activating the adaptive immune response [12,15,26]. Initial recognition of cryptococcal cells by DCs occurs in the lungs. However, cryptococcal cells have a capsule composed of galactoxylomannan (GalXM) and glucuronoxylomannan (GXM). These components have an anti-phagocytic influence on phagocytes, allowing them to evade detection [27,28,29,30,31]. Opsonization by a complement or by anti-capsular monoclonal antibodies negates the anti-phagocytic ability of the polysaccharide capsule, allowing DCs to engulf cryptococcal cells [32,33]. In addition to conventional DCs, plasmacytoid DCs (pDCs) are a rare population of peripheral blood mononuclear cells (PBMCs) found in the blood circulation [34], and these cells also interact with *C. neoformans* [35]. 

### 2.1. Recognition and Activation

Pattern recognition receptors (PRRs) initiate all the fundamental functions of DCs during microbial infections. Found on the cell surface and internally, PRRs are designed to recognize a corresponding set of molecular patterns or microbial pathogen-associated molecular patterns (PAMPs) released by damaged tissues. Association of the PPRs with their PAMPs triggers a cascade of signaling, resulting in the activation of DCs, along with other functions involved in microbial immunity [36,37]. Several studies have examined the role of PRRs during DC recognition and activation by *C. neoformans* [35,38,39,40,41,42,43,44]. Toll-like receptors (TLRs) are PRRs that are expressed on the surface of cells or on the endosomal membrane. During cryptococcal infections, both TLR2 and TLR4 are active, and with the aid of CD14 and CD11/18, bind to polysaccharides in the cryptococcal capsule [38,40]. The importance of these two receptors for anti-cryptococcal immunity has been debated [38,40,45]. TLR2 was shown in vivo to have a role in the survival of mice during infection with the *C. neoformans* strain H99 [46]. However, other studies have found that neither TLR2 nor TLR4 are essential for protective responses, despite their ability to recognize components of the cryptococcal capsule [38,39,40]. While TLR2 and TLR4 were not required, the adaptor molecule MyD88 was required, pointing to the involvement of signaling by some other TLR [38]. TLR2^−/−^ and TLR4^−/−^ mice had similar survival rates and expression of TNF-α, interleukin 1β (IL-1β), IL-12p40, and IL-6 compared to wild-type (WT) mice [38,40]. However, numerous studies have identified TLR9 as necessary for host immunity against the fungal pathogen [40,43,46,47,48,49]. TLR9 is capable of activating myeloid DCs through the recognition of URA5 *C. neoformans* DNA [50,51]. Traditionally, TLR9 is associated with promoting a Th1 protective response and decreasing Th2 hallmarks [48]. Mice deficient in TLR9 were shown to be unable to control cryptococcal infections due to an impairment in cytokine production, which results in an altered adaptive immune response [40,43,49]. This mechanism of impairment is due to a reduction in DC activation in TLR9^−/−^ mice, indicating that TLR9 is important for the activation of DCs and clearance of the cryptococcal cells [38,43,45,46,47,49]. 

**Figure 1:**
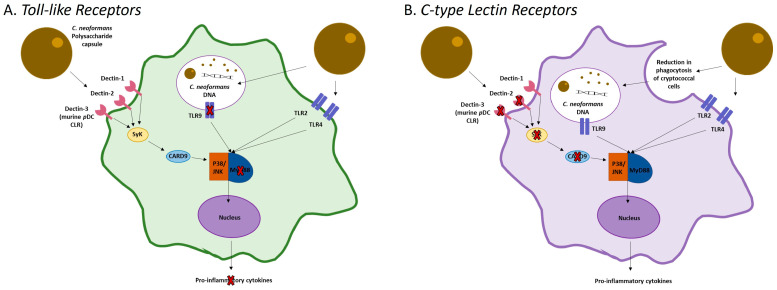
PRRs and CLRs Involved in Cryptococcal Recognition by DCs. DCs are involved in the recognition, engulfment, and killing of pathogens. Initial recognition involves the use of either intra- or extracellular pattern recognition receptors (PPRs), as well as C-type lectin receptors (CLRs). After recognition of their corresponding PAMPS, a cascade of signaling is triggered to initiate the maturation of the DCs and CD-mediated T cell activation. (**A**) While not all TLRs are required for the development of a protective immune response against *C. neoformans*, the intracellular TLR9 is required. TLR9 recognizes URA5 *C. neoformans* DNA after degradation of an engulfed cryptococcal cell. It then initiates a signal cascade involving the adaptor molecule MyD88, which is also a requirement for the initiation of DC maturation and a protective response. (**B**) While not as well studied, some CLRs have been shown to be necessary for the recognition and phagocytosis of *C. neoformans* by DCs. Without the presence of Dectin-2, the signaling molecule SyK, or the adaptor molecule CARD9, there is a reduction in the phagocytosis of cryptococcal cells by DCs. Within pDCs, the requirement of Dectin-3 is dependent on the host.

In addition to TLRs, C-type lectin receptors (CLRs) are involved in the recognition of fungal pathogens and recruitment of DCs (reviewed in [52]) [53]. CLRs are molecules with the capability to detect the polysaccharides within the *C. neoformans* polysaccharide-enriched cell wall and capsule, which is suggested to play a role in the recognition of the fungus by the host’s immune system [53,54]. While the literature has not identified specific CLRs that DCs use for recognition of *C. neoformans*, Dectin-2 is important in the production of cytokines by DCs in response to cryptococcal infections in vivo and ex vivo [41,42,44,53]. Mice lacking Dectin-2 or the adaptor molecule that induces signaling by CLRs, caspase recruitment domain-containing protein 9 (CARD9), displayed a reduction in *C. neoformans* uptake by DCs [53]. However, in another study, Dectin-2 was shown to have no effect on the control of the fungal burden by DCs [41]. Interestingly, the signaling molecules SyK and PI3K were shown to have involvement in DC phagocytosis of the fungal cells. When SyK, a molecule involved in activation by CLR, and a PI3K inhibitor were inhibited, there was a reduction in phagocytosis by BMDCs similar to Dectin-2 knockouts and CARD9 knockout mice [55,56]. Additionally, inhibition of the molecule PI3K resulted in complete abrogation of cryptococcal phagocytosis by BMDCs, indicating that it is essential for DCs to phagocytose *C. neoformans* [55,56]. Moreover, Dectin-3 is required for the recognition and phagocytosis of cryptococcal cells by pDCs, and this is dependent on the host species the cell is derived from. In human pDCs, Dectin-3 is only required for inhibition of cryptococcal growth but is not required for the uptake of cryptococcal cells [35]. Recognition of *C. neoformans* by DCs is summarized in Figure 1. 

After the uptake of cryptococcal cells by DCs through a zipper phagocytosis method, the fungal cell enters the endolysosomal pathway and acquires a lysosomal marker LAMP-1^+^. There, they are killed through both oxidative and non-oxidative methods [12,32,57,58]. Several in vitro studies examining lysosomal extract have shown it has a direct antifungal activity against *C. neoformans* [12,18,58]. However, the exact mechanism and lysosomal components involved in the antifungal activity are not completely known. There is evidence that cathepsin B, a component of the lysosomal extract, possesses the ability to kill *C. neoformans* through the osmotic lysis of cryptococcal cells via damage to their cell wall [58]. Additionally, lysosomal components are being studied in vitro, and six antifungal components (coronin, NOSTRIN, MPO, MMP25, and HNE) have been identified as having antifungal activity in a dose-dependent manner [18]. In addition to conventional DCs, pDCs can also interact with *C. neoformans*, but little is known about their role in the host’s protective immune response against *Cryptococcus*. Both murine and human pDCs have been studied and have displayed the ability to inhibit the growth of cryptococcal cells [35]. pDCs have demonstrated anticryptococcal activity by reactive oxygen species (ROS), which is dependent on recognition by Dectin-3 [35].

### 2.2. Inhibition of DC Maturation

Although they are not the only innate immune cell with the capability to activate T cells, DCs are one of the most proficient, requiring only a select few for the activation of naïve T cells, initiating the adaptive immune response [59]. However, to accomplish this process of DC-mediated T cell activation, the cells must undergo maturation through the increased surface presentation of MHC-II and co-stimulatory molecules (CD80 and CD86) [37,60,61,62]. As mentioned before, GXM has suppressive effects on the host immune response and prevents the phagocytosis of cryptococcal cells, and it non-specifically downregulates T cell proliferation [27]. Furthermore, interactions with cryptococcal mannoproteins can prevent the maturation of DCs in encapsulated strains [28,31,63]. Without proper maturation (increased MHC-II and co-stimulatory molecules), DCs cannot induce a T cell response for proper clearance of the pathogen. With opsonization by a complement or an anti-capsular antibody, DCs are able to recognize *C. neoformans* through CD32 (FcγRII) and CD16 (FcγRIII) to negate these effects [15,31,59]. However, the presence of the capsule is not the only component involved in inhibition of DC maturation. In three acapsular cryptococcal mutants, it was shown that they cause variable stimulation of DC maturation. The cap10 strain was incapable of inducing DC maturation, while both the cap59 and cap67 strains were able to induce the expression of the surface molecule MHC-II and co-stimulatory markers CD86 and CD80 after uptake [63]. However, the cap59 mutant lost its ability to induce DC maturation after incubation with WT *C. neoformans*, which suggests that intact GXM is not required for DC maturation to be inhibited [63,64]. This is a result of the association of glucuronoxylomannan present on the WT cells with the cell surface of the acapsular mutant cap59, preventing DC activation [63]. 

### 2.3. Pulmonary DC Interactions with C. neoformans

As *C. neoformans* typically enters through the respiratory tract, the DCs in the lungs are most important in fungal recognition and control. Multiple pulmonary conventional DC subsets in both human and murine lungs have been identified by surface markers [65,66,67,68,69,70,71,72]. Through this process, three subsets of human pulmonary conventional DCs (CD207^+^, CD14^+^CD1c^+^, and CD14^−^CD1c^+^) and two subsets of murine pulmonary conventional DCs (CD103^+^ and CD11b^+^/monocyte-derived DCs or moDCs) have been identified and profiled. All identified human DCs have been identified as possessing the ability to phagocytose and actively kill cryptococcal cells ex vivo. Both subsets of murine conventional DCs have been shown to interact with cryptococcal cells but have different interactions with *C. neoformans* ex vivo. Neither subset of murine pulmonary DCs was shown to actively kill cryptococcal cells. Interestingly, male CD11b^+^/moDCs were identified as being capable of enhancing the cellular growth of *C. neoformans* ex vivo. [73]. Interestingly, the CD11b^+^/moDCs were shown to infiltrate to the lungs during cryptococcal infection via trafficking by CCR2, and in CCR2-deficient mice, cryptococcal infection led to a shift to Th2 type responses, including increased collagen deposition and increased IL-4, indicating a non-protective immune response [74]. 

As previously stated, DCs have the capability to uptake and kill cryptococcal cells, which allows for the maturation of the DCs and the activation of the adaptive immune system. However, not all species of *Cryptococcus* induce DC-mediated T cell activation [37]. Although *Cryptococcus gattii* is genetically similar to *C. neoformans*, this organism interacts differently with immune cells. Due to their unique capsule, the *Cryptococcus gattii* strain R265 has the ability to suppress human DC-mediated T cell activation despite phagocytosis and cryptococcal killing by the DCs [37,75]. After phagocytosing *C. gattii* R265, the DCs fail to upregulate surface markers such as CD83, CD32, CD86, and MR, which are associated with DC maturation [75]. 

## 3. Macrophages

Macrophages are a heterogenous group of immune cells that are either tissue residents or recruited and interact with *C. neoformans*. They can act either as antifungal cells or they can allow intracellular growth and replication of *C. neoformans* cells. As we will discuss, these outcomes rely on multiple factors, such as macrophage activation status as well as the macrophage subset.

### 3.1. Activation

Macrophage polarization is a continual balance of altering the phenotypes with different functions from tissue repair to antimicrobial activity to maintain and protect the human host from invading pathogens [76]. The gene expression profiles of macrophages have a dynamic flexibility that allows them to alter their activation phenotype based on the changes within their environment [77,78,79,80,81,82,83,84]. As a long-living and self-sustainable resident of the lung alveolar airspaces, the ability of macrophages to be adaptable to their environment is critical in providing protection against invading pulmonary pathogens, such as *C. neoformans*. Otherwise, they would not be able to adjust to various conditions [77,84]. Macrophages can repolarize within hours in response to a different invading pathogen and immune cell-derived signals such as cytokines [82]. 

Polarization bias of macrophages is influenced by cytokine production, secreted cell byproducts, and extracellular expression of receptors. Macrophage phenotypes are broadly classified as either classical (M1) or alternative (M2), with each type having specific functions during the immune response [81,85,86,87]. In addition, the macrophage phenotype is plastic and can change with the local cytokine microenvironment [82,88]. The capability of a macrophage to control cryptococcal growth during infection is reliant on the predominant type of macrophage activation [17,82,89,90,91,92,93]. Polarization of macrophages between the two phenotypes is associated with a Th1- or Th2-dominated adaptive immune response. During a Th1-type immune response, combined with elevated levels of gamma interferon (IFN-γ), macrophages shift predominately towards an M1 phenotype [86]. M1 macrophages are associated with a reduction in the fungal burden and enhanced fungicidal activity through the production of nitric oxide (NO) and reactive oxygen species (ROS) [82,86,94]. Activation of the STAT1-mediated signaling pathway is required for the production of NO by M1 macrophages. In STAT1 KO mice and STAT1 conditional KO mice infected with IFN-γ-producing *C. neoformans*, the mice had an enhanced fungal burden, an enhanced M2 macrophage activation, and a reduction in fungicidal activity when compared to WT mice [95,96,97,98]. STAT1-deficient mice favored *Arg1* production compared to inducible nitric oxide synthase (iNOS) production (*NOS2*), an indicator of M2 macrophage activation [97]. 

While the key initiator of macrophage polarization during *C. neoformans* infections is based on the fluctuation of Th1 and Th2 cytokines in the microenvironment, interactions with *Cryptococcus* may also influence the macrophage polarization state [89,99]. In vitro, *C. neoformans* was shown to suppress NO production by inhibiting *NOS2* expression, resulting in the induction of an M2 macrophage-like state [100,101]. NO is one of the effector molecules involved in anti-cryptococcal activity of M1 macrophages [17,20,90,92,93,95,96,97,102,103,104,105,106]. In both iNOS-deficient mice and iNOS-inhibited WT mice, there was an inability to control intracellular growth of *C. neoformans* within macrophages. This effect remained even in the presence of ROS production [97]. 

Changes in the gene expression of *Cryptococcus*-infected macrophages have been extensively studied. However, differences in gene expression are seen between studies. This may potentially be due to the use of various cell lines versus primary cells [19,73,98,107,108,109]. Recent data revealed the ability of *C. neoformans* to affect the polarization bias of RAW 264.7 macrophages, a murine–leukemia macrophage-like cell line. These cells experienced alterations in genes associated with both lysosomal function and phagocytosis, shifting the polarization of the cells toward a more naïve M0-like state [107]. Additionally, in vivo live *C. neoformans* cells after uptake by murine alveolar macrophages could cause impairment of the lysosome, which can promote an increase in cell proliferation within the phagocyte [88]. During *C. neoformans* infections, the lysosomes of these cells were shown to have fragmented phagolysosomal membranes that become permeable to macromolecules [110,111]. *C. neoformans* can also lead to production of actin flashes outside the phagosome, which presumably prevents the organism from being expulsed from the macrophage [112]. *Cryptococcus*-induced permeabilization of the phagolysosomal membrane is an indicator of lysosomal damage and results in the leakage of phagolysosomal contents into the cytosol of the immune cell [110,111,113]. This leakage has previously been associated with the induction of apoptosis, as well as a loss of antimicrobial contents of the phagolysosome [114]. However, this effect was shown to be negated by the introduction of IFN-γ to the macrophages during their interaction with the cryptococcal cells, allowing the immune cells to maintain fungicidal activity to *C. neoformans* [88,91]. This suggests that an intact phagolysosomal membrane is vital in determining the outcome of a *Cryptococcus* and pulmonary macrophage interaction [88,113].

### 3.2. Trafficking of C. neoformans

In contrast to M1 macrophages, M2 macrophages are associated with being the primary host cell involved in intracellular growth of *C. neoformans* [90,93,106,115]. After uptake, cryptococci traffic in the phagosome, which fuses with the lysosome to become a phagolysosome, results in their exposure to NO, ROS, degradative enzymes, and an acidic environment [116,117]. *C. neoformans* are shown to be capable of hindering acidification of the phagosome, allowing for intracellular proliferation in both human monocyte-derived macrophages and in the J774A.1 macrophage cell line [118]. However, *C. neoformans* can also survive in an acidic environment [117]. Additional cryptococcal proteins are required for intracellular growth to occur. Replication within macrophages requires the presence of phospholipase B (*PLB1*), a known factor of *Cryptococcus* virulence. Deletion of the gene *PLB1* in *C. neoformans* led to reduced survival and replication in macrophages [119,120]. Additionally, F-box protein 1 (*fb1*) and its substrate inositol phosphosphingolipid-phospholipase C1 are necessary for the spread of the pathogen to the central nervous system (CNS) and for resistance to NO [121]. Following M1 polarization by stimulation of IFN-γ, the fungal pathogens’ ability to induce lysosomal damage is negated and the macrophages exhibit an increase in fungal killing [88]. 

In addition to intracellular growth, *C. neoformans* cells are capable of non-lytic exocytosis from macrophages through the escape of cryptococci from the phagocyte without destroying the immune cell in the process (in vitro and in vivo models [7,122,123]). Both cryptococcal virulence factors and host factors regulate non-lytic exocytosis of the cells [123,124]. The capability of the fungal pathogen to successfully survive and replicate within the host immune cells contributes to increased fungal dissemination within the host. It has been suggested that the macrophages act as a “Trojan Horse” during cryptococcal pathogenesis, carrying the pathogen across the blood–brain barrier (BBB) by trans-endothelial pores [125,126]. Once the fungal organism crosses the BBB, it can lead to the development of a CNS infection and meningoencephalitis [126,127,128,129]. Unfortunately, this infection is extremely dangerous, with survivors often developing adverse effects including neurological deficits [130,131]. After invasion of the CNS, the microglial response is essential against the invading *C. neoformans*. Microglia are brain-resident macrophages and are found throughout the parenchyma of the brain. Similar to other innate immune cells, they possess two states. In a healthy host, they remain in a resting state until activation [132]. Upon activation, they change their overall shape into an amoeboid-like morphology, which allows them to phagocytose pathogens, infected cells, and dead neurons [133,134,135,136,137,138]. These cells can recognize fungal PAMPs via TLRs. Recognition of fungal PAMPs promotes the release of antimicrobial molecules and proinflammatory cytokines into the microenvironment for the recruitment of innate and adaptive immune cells [5,139]. Interestingly, there are differences between murine and human microglia, with human cells being unable to kill *C. neoformans* [140]. Since the secretion of NO is positively correlated with the cells’ ability to kill *C. neoformans*, it is potentially due to the inability of human microglia to produce a sufficient amount of NO compared to the murine microglia [140,141,142]. 

Dissemination to other organs, such as the liver, can also occur during cryptococcosis. However, macrophages resident to the liver, Kupffer cells (KCs), are able to modulate liver infection by *Cryptococcus* [143]. Phagocytosis by KC involves complement receptors CR3 and CRIg and scavenger receptors, and inhibition of *Cryptococcus* growth is dependent on IFN-γ but not on IFNγR signaling [143]. 

Ly6c^+^-expressing inflammatory monocytes (IFM) are precursors to both macrophages and DCs and, like their derivatives, they are able to inhibit the growth of cryptococcal cells [74,144,145,146,147,148,149,150]. However, despite their ability to inhibit cryptococcal growth, they may also be involved in the progression of *C. neoformans* infections. When IFMs were ablated in vivo, there was an improvement in the fungal burden. A transcriptional analysis of the IFMs identified M2 surface marker expression when challenged by *C. neoformans* [144]. M2 macrophages are associated with host tissue repair and homeostasis. They are not typically associated with inflammatory-type cells [151]. However, the M2 macrophage phenotype is shown to be involved in the intracellular growth of *C. neoformans* [90,93,106,115].

### 3.3. Pulmonary Macrophage Interactions with C. neoformans

Since *C. neoformans* is typically inhaled, it is important to investigate the role of pulmonary macrophages. The pulmonary macrophages consist of a diverse population of cells [65,152,153,154]. Originally, it was assumed in the pulmonary region that macrophage populations were divided into alveolar macrophages (AM) and interstitial macrophages (IM). Each was named for their respective designated region of the pulmonary airways and tissues [155]. More recently, however, multiple pulmonary macrophage subsets in both human and murine lungs have been identified by flow cytometry [65,73,108], and their lineages have been defined by lineage tracing [66,68,156,157,158,159,160]. Through this process, three subsets of human pulmonary macrophages (AM, CD14^+^CD1c^−^, and CD14^−^CD1c^−^) have been identified and profiled in healthy human lungs [65]. All human subsets internalize cryptococci, although AMs and CD14^+^ macrophages are more efficient at fungal uptake compared to CD14^−^ macrophages [108]. Furthermore, following interactions with *Cryptococcus*, the fate of the pathogen varied between subsets [108]. While AMs displayed consistent antifungal activity against the fungal pathogen, CD14^−^ and CD14^+^ macrophages were unable to kill cryptococcal cells after uptake [108]. Transcriptional analyses of these subsets following interaction with *C. neoformans* compared to the subsets alone revealed changes in gene expression within metabolism (MTRNR2L12, MT-ND6, MT-ATP8, MT-CO_3_, and MT-CYB) and antigen presentation genes (HLA-A, HLA-B, HLA-C, and HLA-DRA) [108]. Additionally, four murine macrophage subsets (AM, IM, Ly6c^+^, and Ly6c^−^ monocyte-like macrophages) have been identified through flow cytometry [73], as well as gene expression profiling and lineage tracing [66,67,68,70,72,157,159,160,161,162]. All identified murine macrophage subsets can interact with cryptococcal cells, but only female Ly6c^-^ monocyte-like macrophages can significantly inhibit the cellular growth of cryptococcal cells. These cells expressed a significant upregulation in MHC-I and significant regulation in several metabolic genes [73]. The MHC-I antigen presentation pathway is associated with the initiation of the adaptive immune response to virally infected cells, but can also be involved in cross-presentation of cryptococcal antigens [163,164,165].

## 4. Neutrophils

Neutrophils or polymorphonuclear leukocytes (PMNs) are phagocytes that are recruited during cryptococcal infections to the lungs [9,166,167,168]. They are one of the most abundant types of immune cells present in the human bloodstream and stem from the bone marrow in large amounts of ~10^11^ cells per day [169]. These cells are essential in the killing and regulation of cryptococcal cells during the initial infection and are shown to have antifungal abilities greater than monocytes and macrophages [115,170,171]. After infection, circulating neutrophils migrate to infection sites and aid in fungal clearance. They can kill using both intracellular and extracellular methods, and by oxidative and non-oxidative mechanisms [9]. Neutrophil swarming is essential for the innate immune cells to accumulate at sites of infection for the clearance of pathogens [172]. In vivo, this process of neutrophil migration is mediated by the presence of complement C3 and C5a-C5aR complement pathways and actin polymerization to the fungal infection site [173,174]. Complement C3 is required for neutrophil swarming to cryptococcal cells. In C3^−/−^ murine models, no interaction between *C. neoformans* and neutrophils occurred despite the presence of the cryptococcal polysaccharide capsule [174]. This is likely due to a lack of complement C3b opsonization of the fungal cells [175]. 

C5a-C5aR is important in fungal clearance in three ways. First, C5aR significantly increased the distance they can travel [176]. Secondly, C5aR also enhances the expression of the surface marker CD11b. CD11b is a part of the integrin Mac-1 molecule, a monocyte, neutrophil, and macrophage surface receptor, and along with CD18, forms complement receptor 3 [177,178]. CD11b helps with the adhesion of these phagocytes to various cells and regulates antimicrobial responses, including phagocytosis and migration [178]. Mac-1 binds to its ligand ICAM-1 during cryptococcal infections. Blockage of CD11b almost completely abolishes neutrophil ability to kill cryptococcal cells by preventing the cells from phagocytosing the fungus [176]. Additionally, CD11b is critical for neutrophil transmigration through endothelial cells. CD11b-knockout neutrophils were shown to be defective in intravascular crawling, and the majority failed to go through transendothelial migration [179,180]. Lastly, C5aR signaling mediates the migration of neutrophils to *C. neoformans*. Interestingly, C5a is not completely required for the swarming of neutrophils around *C*. *neoformans* cells [173,176]. While C5^−/−^ mice were shown to have fewer neutrophil clusters compared to WT mice, there was still a presence of neutrophil clustering around *C. neoformans*. Despite the lack of C5a-C5aR signaling, *C. neoformans* are still able to be opsonized by C3b/iC3b [175]. However, C5a is required for the ideal killing of the fungus by neutrophils via the formation of a concentration gradient around encapsulated cryptococcal cells [176]. Further studies have showed that the mitogen-activated protein kinase (MAPK) pathway is involved in the migration of neutrophils using the C5a-C5aR complement pathway. Inhibition of the p38 kinase resulted in a decrease in neutrophil-mediated cryptococcal killing and migration [173,181,182,183]. 

During an infection, neutrophils in close proximity to the infection site are initially recruited via C5a-C5aR signaling as discussed above [184]. Once this occurs, chemotactic neutrophils will secrete leukotriene B4 (LTB4), creating a second chemical gradient that increases the range of the primary chemoattractant [175,185]. When LTB4 synthesis is inhibited in vivo, there is no formation of large neutrophil clusters and there is a significant reduction in neutrophil migration to the lungs, indicating that LTB4 is not only required for the huge migration of distant neutrophils but also for the swarming of neutrophils within the lungs [175,186]. 

*C. neoformans* can also influence the neutrophil fungicidal response. Neutrophils can combat invading pathogens by releasing a neutrophil extracellular trap (NET), made up of condensed chromatin with cytosolic and granular proteins [187,188]. These NETs retain and kill the microbe, while protecting the host cells. *C. neoformans* can inhibit NET production with a component of the capsule, glucuronoxylomannan (GXM) [187]. Cryptococcal capsular components can also inhibit the migration of neutrophils to the infection site. They prevent neutrophil migration by releasing components of their capsule, which interferes with trafficking due to chemokine gradients and neutrophil rolling [189]. 

Circulating neutrophils are capable of interaction with *C. neoformans* cells arrested to walls of blood vessels. After engulfment of the adhered cells, they can enter back into the bloodstream with the fungal cell, resulting in the removal of cryptococcal cells from the brain vasculature [23,176,190]. Clearance of fungal cells within the brain vasculature is mainly facilitated by the neutrophils. However, the efficiency of neutrophils in the intravascular clearance of *C. neoformans* in the brain is significantly lower than in the pulmonary region of the human body [176]. 

Despite neutrophils being capable of removing and killing *C. neoformans*, their role in cryptococcal infections is still largely unknown. In vivo studies have presented controversial results, portraying neutrophils as having either protective or damaging roles [115,191]. During the protective immune response, depletion of neutrophils does not affect the fungal burden in pulmonary tissue of mice [16,192], and neutropenic mice survived longer after the initiation of a pulmonary *C. neoformans* infection than those with normal neutrophil counts [17,193]. In HIV patients with cryptococcal meningitis, an increased neutrophil count was associated with a higher mortality rate [193]. This suggests that neutrophils are not required for the clearance of *C. neoformans* by the host’s immune system and may actually aid in cryptococcal pathogenesis [16,17,192]. However, the detrimental role of neutrophils during cryptococcal infections is still under debate as the depletion of neutrophils has also been associated with a reduction in fungal clearance in the brain [23]. 

*C. neoformans* is a known facultative intracellular pathogen of monocyte-derived macrophages and dendritic cells [110,111,118,194]. However, little is known about the ability of *C. neoformans* to survive within neutrophils after phagocytosis [23]. Non-lytic exocytosis of *C. neoformans* has previously been seen in macrophages and monocytes and has long been suspected to contribute to the spread of fungal cells in the neural tissue of the host, resulting in cryptococcal meningitis [123,195,196,197]. However, these innate immune cells are not the only cells involved in the potential trafficking of *C. neoformans* across the blood–brain barrier. Neutrophils, despite their vital role in the innate immune response, have demonstrated similar behaviors [24]. Neutrophils were shown to both phagocytose and traffic cryptococcal cells to the brain and deposit them into the brain vascular tissue by exocytosis. This may contribute to brain infections and may explain why the fungal burden in the brain decreased in correlation with a reduction in neutrophils present in the blood [23]. 

## 5. Conclusions

**Figure 2:**
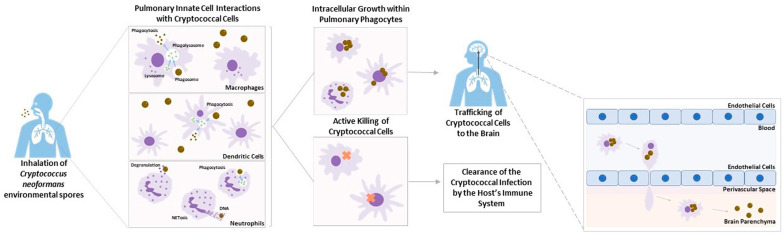
Two Distinct Outcomes of Interactions with Human Innate Phagocytes. Following recognition of cryptococcal cells within the alveoli of the lungs by the host’s pulmonary phagocytes, the innate immune cells are involved in the removal of the pathogen and clearance of the cryptococcal infection. There are two distinct outcomes of the cryptococcal–host interaction. Once *C. neoformans* cells have been internalized, some cells are capable of actively killing the fungal cells, resulting in clearance of the cryptococcal infection from the host. However, a few cell types/subsets have been shown to be permissive to cryptococcal growth, allowing for intracellular growth and ultimately the trafficking of cryptococcal cells to the central nervous system. These cells are able to transport the fungal cells across the blood–brain barrier (BBB), resulting in the development of cryptococcal meningitis.

Innate immune cells are the first to interact with invading pathogens. They aid in preventing the establishment of infections and their potential dissemination from the primary site of infection. While innate immune phagocytes are known to have important roles in the identification and removal of pathogens such as *C. neoformans*, they can also play a role in cryptococcal pathogenesis. As summarized in Figure 2, clearance of the pathogen by these cells is not always the outcome of the innate immune phagocyte interaction. Recent studies have found both beneficial and damaging abilities of these innate immune cells during cryptococcal pathogenesis. *C. neoformans* has mechanisms that can interfere with the ability of host phagocytes to recognize, phagocytose, and clear fungi during infection, allowing for the dissemination of the organism to the CNS. Though mechanisms of host evasion by the fungal pathogen have been extensively studied over the years, there is still more to learn, specifically regarding the ability of the microorganism to use the host’s innate immune cells as transportation to extra-pulmonary regions of the human body.
